# Oral pharmacokinetics and efficacy of oral phospholipid remdesivir nucleoside prodrugs against SARS-CoV-2 in mice

**DOI:** 10.1128/aac.01039-24

**Published:** 2024-09-06

**Authors:** Aaron F. Carlin, James R. Beadle, Jeremy Ardanuy, Alex E. Clark, Victoria Rhodes, Aaron F. Garretson, Joyce A. Murphy, Nadejda Valiaeva, Robert T. Schooley, Matthew B. Frieman, Karl Y. Hostetler

**Affiliations:** 1 Department of Pathology, University of California San Diego, La Jolla, California, USA; 2 Department of Medicine, University of California San Diego, La Jolla, California, USA; 3 Department of Microbiology and Immunology, The University of Maryland School of Medicine, Baltimore, Maryland, USA; 4 Center for Pathogen Research, The University of Maryland School of Medicine, Baltimore, Maryland, USA; IrsiCaixa Institut de Recerca de la Sida, Barcelona, Spain

**Keywords:** antiviral agent, broad spectrum antiviral, lipid prodrug, remdesivir, remdesivir nucleoside, SARS-CoV-2, COVID-19, mouse model, *in vivo* efficacy, pharmacokinetics

## Abstract

Oral broad-spectrum antivirals are urgently needed for the treatment of many emerging and contemporary RNA viruses. We previously synthesized 1-*O*-octadecyl-2-*O*-benzyl-*sn*-glyceryl-P-RVn (ODBG-P-RVn, V2043), a phospholipid prodrug of GS-441524 (remdesivir nucleoside, RVn), and demonstrated its *in vivo* efficacy in a SARS-CoV-2 mouse model. Structure-activity relationship studies focusing on the prodrug scaffold identified two modifications, 3-fluoro-4-methoxy-benzyl (V2053) and 4-cyano-benzyl (V2067), that significantly enhanced the *in vitro* broad-spectrum antiviral activity against multiple RNA viruses when compared to V2043. Here, we demonstrate that V2043, V2053, and V2067 are all orally bioavailable, well-tolerated, and achieve high sustained plasma levels after single oral daily dosing. All three phospholipid prodrugs are significantly more active than RVn *in vitro* and significantly reduce SARS-CoV-2 lung titers in prophylaxis and treatment mouse models of SARS-CoV-2 B.1.351 infection. On a molar basis, V2043 and V2067 are substantially more active than obeldesivir/GS-5245 and molnupiravir *in vivo*. Together, these data support the continued development of phospholipid RVn prodrugs for the treatment of SARS-CoV-2 and other RNA viruses of clinical concern.

## INTRODUCTION

Broad-spectrum potent antivirals are urgently needed to combat contemporary and emerging RNA viruses for which few treatments exist (1). Remdesivir is a potent broad-spectrum inhibitor of the RNA-dependent RNA polymerase of many epidemic and zoonotic RNA viruses, including SARS-CoV-2. Additionally, remdesivir has demonstrated a high barrier to resistance both *in vitro* and in human clinical studies (2–4). Early administration of remdesivir to non-hospitalized high-risk individuals decreased the risk of hospitalization or death by 87% (5). However, the requirement to administer remdesivir intravenously severely limits its use early in the disease when it is most effective and in individuals with limited access to health care. Thus, the development of oral drugs that produce high levels of remdesivir triphosphate, the active metabolite, in lung tissue is a high priority for the treatment of respiratory RNA viruses.

**Fig 1:**
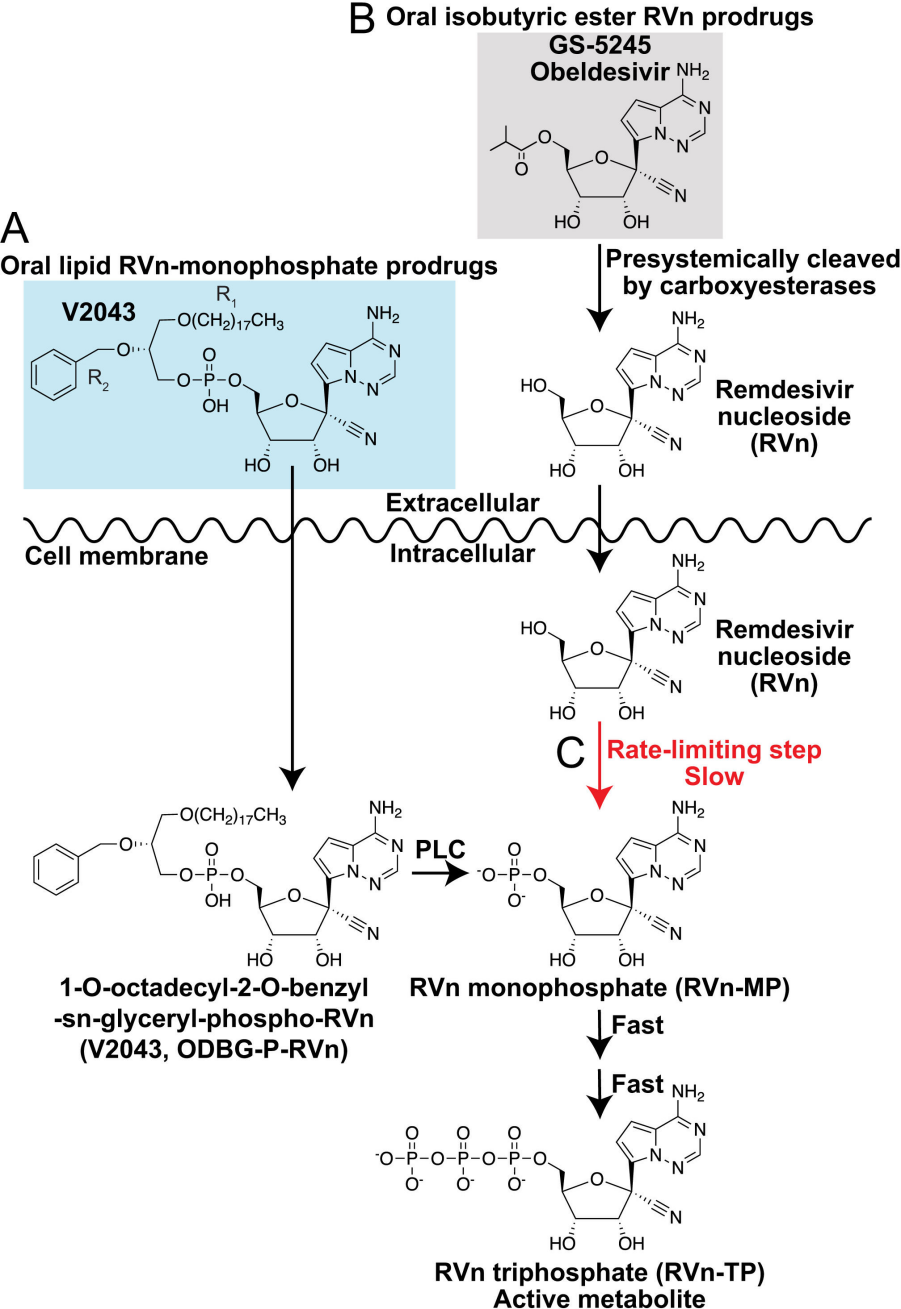
Phospholipid RVn prodrugs bypass rate-limiting phosphorylation. Metabolic activation pathways of lipid RVn monophosphate prodrugs and isobutyric acid RVn prodrugs, such as GS-5245. (**A**) Orally administered lipid RVn-MP prodrugs are absorbed, distributed, and taken up into cells intact. Intracellularly, these compounds are directly converted to RVn-MP through phospholipase C cleavage and then metabolized to the active metabolite, RVn-TP, by nucleotide kinases. Bypass of the rate-limiting first phosphorylation step increases the efficiency of lipid RVn-MP prodrug metabolism to RVn-TP. (**B**) Orally administered isobutyryl ester RVn prodrugs are metabolized pre-systemically by carboxyesterases in the intestine and liver to RVn. Circulating RVn is taken up into cells where it is first metabolized to RVn-MP, and then to the active metabolite, RVn-TP by nucleoside kinases. (**C**) Conversion to the active metabolite, RVn-TP, is rate limited by the metabolism of RVn to RVn-MP.

We previously synthesized a potent phospholipid RVn prodrug, 1-*O*-octadecyl-2-*O*-benzyl-*sn*-glyceryl-P-RVn (ODBG-P-RVn, V2043), possessing excellent oral bioavailability and stability in plasma (6). Using structure-activity relationship studies focused on the R_1_ and R_2_ positions of the prodrug scaffold, we identified two modifications that significantly increased the *in vitro* antiviral activity compared to V2043, 3-fluoro-4-methoxy-benzyl (V2053), and 4-cyano-benzyl (V2067) (7, 8). In contrast to isobutyryl ester RVn prodrugs in clinical development, phospholipid RVn prodrugs such as V2043, V2053, and V2067 are absorbed and enter cells intact (Fig. 1). They then undergo phospholipase C catabolism to RVn-monophosphate thereby bypassing rate-limiting kinase activation, which can increase antiviral potency (Fig. 1A). Here, we report the *in vivo* pharmacokinetics of V2043, V2053, and V2067, their relative *in vitro* activity compared to RVn, and their *in vivo* efficacy against SARS-CoV-2.

## RESULTS

### Phospholipid RVn prodrugs are more potent than RVn *in vitro*

The relative *in vitro* activity of V2043, V2053, and V2067 compared to RVn was evaluated using SARS-CoV-2 strain WA1 infections in four cell types. Compared to RVn, all phospholipid RVn prodrugs had significantly lower EC_50_s in the cell types tested (Table 1). This suggests that phospholipid RVn prodrugs undergo rapid internalization and efficient conversion to RVn-triphosphate due, at least in part, to kinase bypass. Additionally, consistent with our previous data, V2053 and V2067 had lower EC_50_s than V2043 in most cell types (7, 8) (Table 1).

**TABLE 1:** Phospholipid RVn prodrugs are more potent inhibitors of SARS-CoV-2 than RVn *in vitro^b^*

Cell line	Compound	R_1_	R_2_	EC_50_ μM ± SD	EC_90_ μM ± SD	CC_50_ μm	SI	Relative activity vs RVn	Significant vs RVn
Human lung epithelial (A2T2-A549) cell line expressing human angiotensin-converting enzyme 2 (ACE2/A2) and transmembrane serine protease 2 (TMPRSS2/T2)	RVn	na	na	3.006 ± 0.038	4.486 ± 0.256	>100	>33	−	−
	Obeldesivir GS-5245	na	na	1.196 ± 0.084	1.888 ± 0.460	−	−	−	−
	V2043	octadecyl	(R)-Bn	0.566 ± 0.028	1.624 ± 0.227	>100	>176	5.3×	*P* < 0.0001
	V2053	oleyl	3-F-4-MeO-Bn	0.410 ± 0.040	0.811 ± 0.059	>100	>243	7.3×	*P* < 0.0001
	V2067	octadecyl	4-CN-Bn	0.213 ± 0.026	0.441 ± 0.071	82.3	386	14.1×	*P* < 0.0001
Human lung epithelial (Calu-3) cell line	RVn	na	na	0.665 ± 0.284	1.892 ± 0.497	>100	>213	−	−
	V2043	octadecyl	(R)-Bn	0.168 ± 0.074	0.484 ± 0.142	58.3^*a*^	486	4.0×	*P* = 0.002
	V2053	oleyl	3-F-4-MeO-Bn	0.074 ± 0.014	0.153 ± 0.047	58.7^*a*^	699	9.0×	*P* < 0.0001
	V2067	octadecyl	4-CN-Bn	0.053 ± 0.018	0.172 ± 0.140	60.2^*a*^	1,338	12.5×	*P* < 0.0001
Human hepatoma (Huh7.5) cell line	RVn	na	na	1.358 ± 0.544	2.957 ± 0.866	>100	74	−	−
	V2043	octadecyl	(R)-Bn	0.117 ± 0.009	0.293 ± 0.042	55.8^*a*^	477	11.6×	*P* = 0.0001
	V2053	oleyl	3-F-4-MeO-Bn	0.047 ± 0.015	0.136 ± 0.058	>100^*a*^	>2,128	28.9×	*P* < 0.0001
	V2067	octadecyl	4-CN-Bn	0.036 ± 0.014	0.096 ± 0.037	47.2^*a*^	1,311	37.7×	*P* < 0.0001
African green monkey (Vero-TMPRSS2) cell line expressing human TMPRSS2	RVn	na	na	1.078 ± 0.264	2.428 ± 0.692	>100	>92	−	−
	V2043	octadecyl	(R)-Bn	0.104 ± 0.030	0.248 ± 0.052	82.6	794	10.4×	*P* < 0.0001
	V2053	oleyl	3-F-4-MeO-Bn	0.134 ± 0.050	0.367 ± 0.139	>100	>746	8.0×	*P* = 0.0001
	V2067	octadecyl	4-CN-Bn	0.046 ± 0.014	0.107 ± 0.020	57.0	1,239	23.4×	*P* < 0.0001

^*a*^ CC_50_ values previously published by our group using identical conditions (7).

^*b*^ EC_50_, 50% effective inhibition concentration; EC_90_, 90% effective inhibition concentration; CC_50_, 50% cytotoxic concentration; SI, selectivity index = CC_50_/EC_50_; Relative activity vs RVN = RVN EC_50_/Test Compound EC_50_. All assays were carried out using SARS-CoV-2 isolate USA-WA1/2020 using immunofluorescence (IF) detection of N protein. Mean values ± standard deviation values were derived from a minimum of at least three independent experiments performed in biological duplicate. CC_50_ values were determined by CellTiter-Glo (maximum concentration 100 mM) after 48 h of incubation. Virus infections were analyzed at 32 (TMPRSS2-vero), 44–48 (calu3), or 48 (huh7.5 and A2T2-A549) hours post-infection (hpi). EC_50_, EC_90_, and CC_50_ values are in micromolar units and were calculated using Graphpad Prism software. Statistical significance compared to RVn was calculated using a one-way ANOVA with Dunnett’s post-test corrected for multiple comparisons. −, not done.

### Oral daily V2043 and V2067 administration achieve therapeutic plasma levels

**Fig 2:**
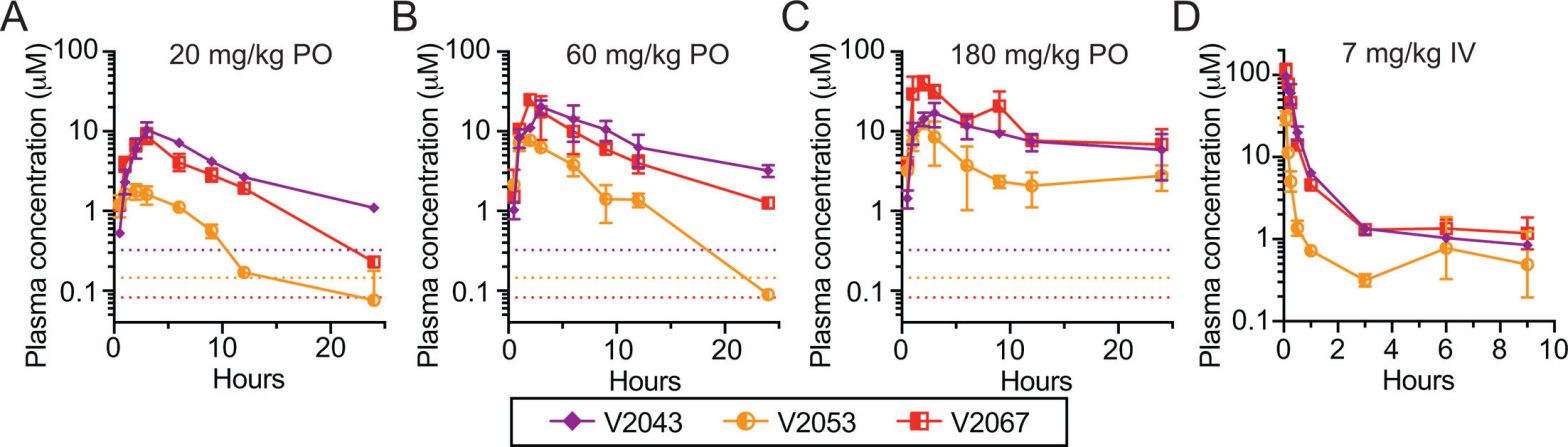
*In vivo* plasma pharmacokinetics of oral phospholipid RVn prodrugs after single dose PO or IV administration in BALB/c mice: (**A–C**) Male 22–24 g BALB/c mice were given a single oral dose of (**A**) 20, (**B**) 60, or (**C**) 180 mg/kg in 0.1 M sodium carbonate/bicarbonate pH 9 and plasma was obtained at 0.5, 1, 2, 3, 6, 9, 12, and 24 h (*In Vivo* Technologies, Moffatt Field, CA). Dotted lines represent the *in vitro* EC_90_ for SARS-CoV-2 infection in Calu-3 cells: V2043 = 0.320 μM, V2053 = 0.154 μM, and V2067 = 0.082 μM in Calu-3 (7). (**D**) Male 20–22 g BALB/c mice were given a single dose 7 mg/kg by slow IV infusion in Ringer’s Lactate pH 7.4 and plasma was obtained at 0.083, 0.16, 0.25, 0.5, 1, 3, 6, and 9 h (*In Vivo* Technologies, Moffatt Field, CA). Plasma samples were analyzed to determine prodrug levels (Aliri Bio, Colorado Springs, CO).

Given the antiviral potency of V2043, V2053, and V2067, we performed single escalating dose oral pharmacokinetic (PK) evaluation of each prodrug at 20 mg/kg, 60 mg/kg, or 180 mg/kg in BALB/c mice. All doses were well tolerated with no appreciable adverse effects. V2043 and V2067 plasma prodrug concentrations exceeded the EC_90_ of SARS-CoV-2 for 24 h post administration at all three doses tested (Fig. 2A through C). In contrast, V2053 plasma levels declined more rapidly, leading to plasma concentrations that fell below the EC_90_ between 12 and 24 h post-administration when given at 20 or 60 mg/kg (Fig. 2A and B). When administered at 60 mg/kg, peak plasma concentrations (Cmax) of V2043, V2053, and V2067 reached 20.3, 7.66, and 24.9 µM, respectively (Table 2). Comparing intravenous (IV) and oral prodrug exposure the oral bioavailability of V2043, V2053, and V2067 was 73.6%, 51%, and 57%, respectively (Fig. 2D; Table 2).

**TABLE 2:** *In vivo* plasma pharmacokinetics of oral phospholipid RVn prodrugs in BALB/c mice^*a*^

Route	Compound	Dose mg/kg	CL/F (L/kg/hours)	Vd/F (L/kg)	*T*1/2 (hours)	*C*max µM	AUC µM*hours	*F*%
IV	V2043	7			0.70	96.58	46.60	−
	V2053	7			0.29	30.22	9.58	−
	V2067	7			0.23	115.51	36.16	−
Oral	V2043	20	0.261	1.68	4.46	10.6	90.1	73.6
	V2053	20	1.68	14.60	6.04	1.78	13.60	51.0
	V2067	20	0.373	2.18	4.06	8.83	64.70	57.0
	V2043	60	0.334	3.04	6.31	20.3	199.00	−
	V2053	60	1.34	6.67	3.44	7.66	53.10	−
	V2067	60	0.45	4.38	6.83	24.90	154.00	−
	V2043	180	0.846	8.61	7.06	17.0	211.00	−
	V2053	180	1.81	18.50	7.06	12.50	90.80	−
	V2067	180	0.49	8.74	12.40	41.00	332.00	−

^*a*^ Mice were treated and sample collected and analyzed as described in Fig. 2. Pharmacokinetic data were provided by Thermo Scientific’s Watson LIMS package (version 75 SP2) using linear trapezoidal fitting to mean overall data. Twelve subjects were used, two time point per subject, three subjects per time point for a total of eight time points. CL/F, apparent oral clearance; Vd/F, volume of distribution; *T*1/2, half life; *C*max, maximum plasma concentration; AUC, area under the curve; *F*%, oral bioavailability. −, not done.

**Fig 3:**
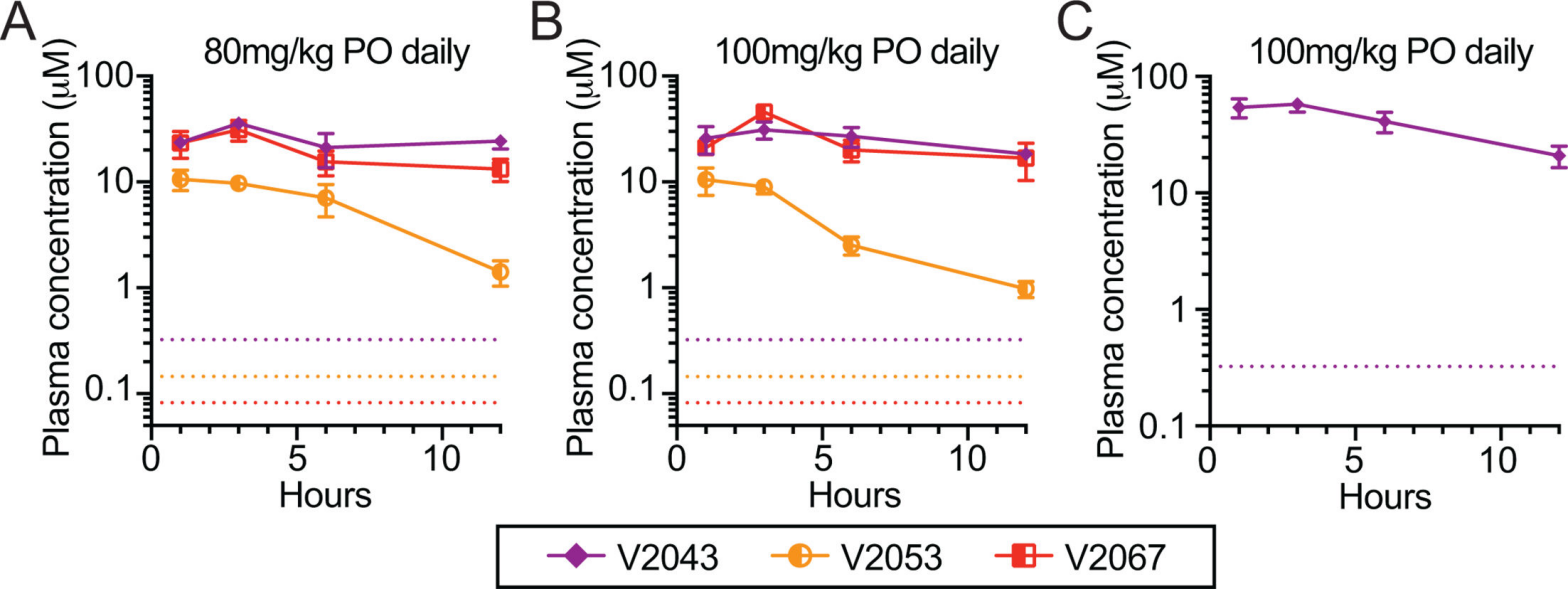
Oral RVn prodrugs maintain high serum levels after repeated dosing in mice and hamsters. Multiple-dose oral tolerability and pharmacokinetics in mice and Syrian hamsters: (**A and B**) 5-day oral tolerability study in BALB/c mice with pharmacokinetics obtained after the final dose. Male 20–22 g mice were given the indicated drugs at (**A**) 80 mg/kg or (**B**) 100 mg/kg PO daily in 0.1 M sodium carbonate/bicarbonate pH 9 for 5 days. Plasma was obtained after the last dose at 1, 3, 6, and 12 h (*In Vivo* Technologies, Moffatt Field, CA). (**C**) 7-day oral tolerability study in Syrian hamsters with pharmacokinetics obtained after the final dose. Male 95–130 g Syrian hamsters were given V2043 at 100 mg/kg PO daily in 0.1 M sodium carbonate/bicarbonate pH 9 for 7 days and plasma was obtained after the last dose at 1, 3, 6, and 12 h (Attentive Sciences, Stillwell, KS). All plasma samples were analyzed to determine prodrug levels (Aliri Bio, Colorado Springs, CO). Dotted lines represent the *in vitro* EC_90_ for SARS-CoV-2 infection in Calu-3 cells: V2043 = 0.320 μM, V2053 = 0.154 μM, and V2067 = 0.082 μM in Calu-3 (7).

To further assess tolerability, BALB/c mice were administered once-daily oral doses of each prodrug for 5 days at concentrations of 80 mg/kg or 100 mg/kg daily (Fig. 3A and B). Both doses were well tolerated without appreciable adverse effects or weight loss (Fig. S1A). All compounds maintained sustained plasma prodrug levels well above the EC_90_ for SARS-CoV-2 following the final dose. Similarly, seven-day 100 mg/kg once-daily oral V2043 in Syrian hamsters was well tolerated without any appreciable toxicity (Fig. 3C**;** Fig. S1B).

### V2043 and V2067 are more potent SARS-CoV-2 inhibitors than GS-5245 in BALB/c mice

**Fig 4:**
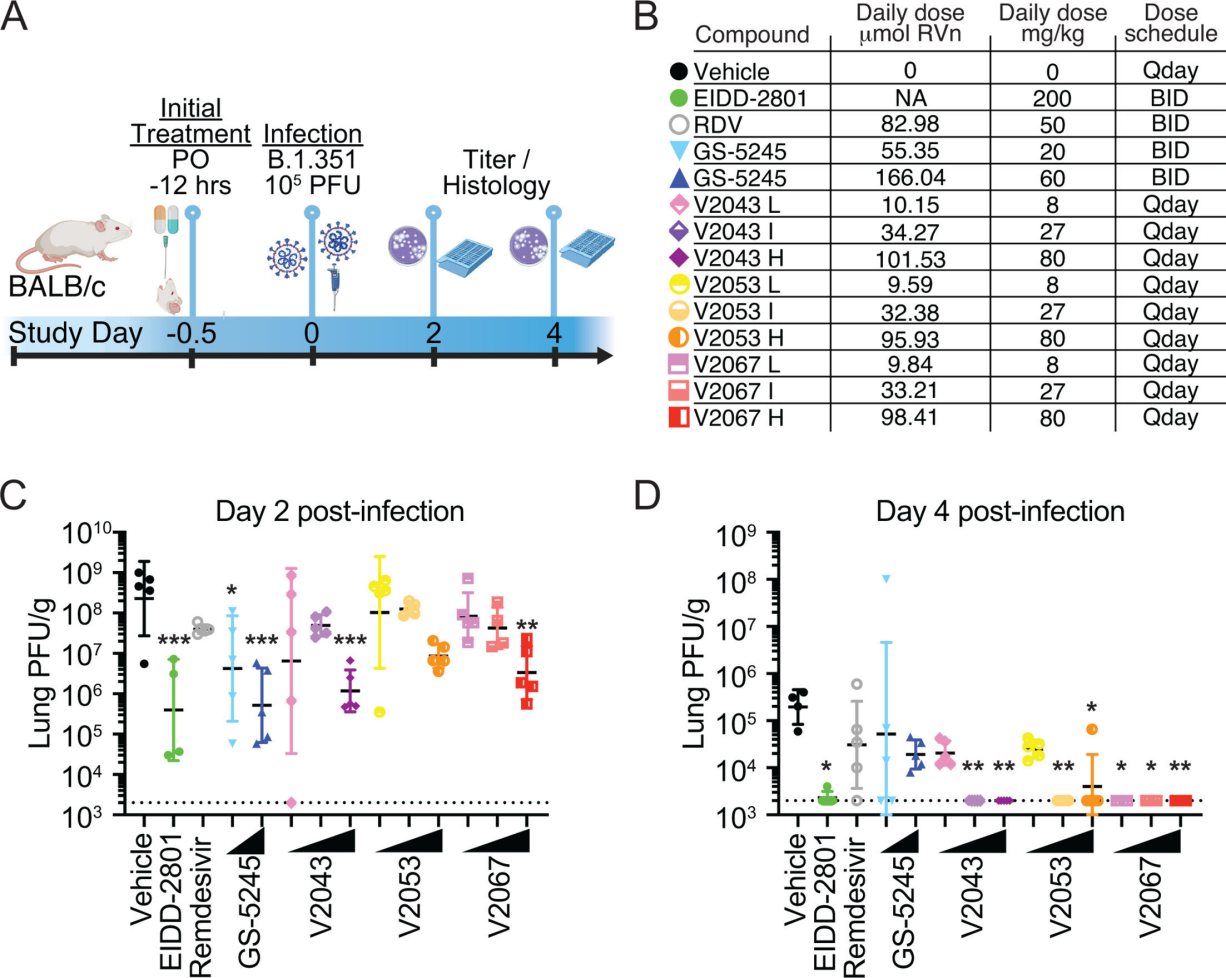
Prophylactic efficacy of oral phospholipid RVn prodrugs in a SARS-CoV-2 mouse model: (**A**) 8- to 10-week-old wild-type Balb/c mice (*n* = 5 per group/time point) were challenged with 1 × 10^5^ PFU of SARS-CoV-2 Beta variant (B.1.351). (**B**) Mice were treated with vehicle, remdesivir (GS-5734), EIDD-2801 (molnupiravir), GS-5245 (obeldesivir), or lipid RVn prodrugs V2043, V2053, or V2067 orally beginning 12 h before infection. (**C and D**) Lungs were harvested at (**C**) day 2 and (**D**) day 4 post-infection for viral titer quantification. *n* = 5 mice per group, mean ± SD is shown. Groups were compared using a mixed effect model with a comparison of each treatment group with vehicle controls using Dunnett’s multiple comparison test; *P < 0.05, **P < 0.01, and ***P < 0.001. Dotted lines represent the PFU limit of detection.

Given the promising *in vitro* potency and *in vivo* PK, we evaluated the *in vivo* efficacy of V2043, V2053, and V2067. We first performed a prophylactic dose escalation study in 8- to 10-week-old female BALB/c mice, in which they were inoculated intranasally with 10^5^ plaque-forming units (PFU) of SARS-CoV-2 B.1.351 (Beta variant) (Fig. 4A). This model typically results in moderate levels of weight loss (5%–15% body weight), high lung viral titers [~10^8^ PFU/g in the lung by 2 days post-infection (dpi)], and substantial lung inflammatory pathology (9). For prophylactic dosing, all compounds were administered starting 12 h prior to infection. Each phospholipid RVn prodrug was orally administered at three doses, 8 mg/kg, 27 mg/kg, or 80 mg/kg once daily (Fig. 4B). As comparators, mice were orally administered EIDD-2801 (molnupiravir) at 100 mg/kg twice-daily (BID), remdesivir (GS-5734) at 25 mg/kg BID, or obeldesivir (GS-5245) at 10 mg/kg or 30 mg/kg BID (Fig. 4B). Lungs were harvested at day 2 or day 4 post-infection for viral titer quantification and histology (Fig. 4A). Weights were measured daily as were symptoms, which were used to determine a clinical score. At 2 dpi, V2043, V2053, and V2067 demonstrated dose-dependent antiviral effects (Fig. 4C). V2043 and V2067 at 80 mg/kg once daily significantly reduced lung titers by 2.29 and 1.83 Log_10_, respectively (Fig. 4C). Similarly, EIDD-2801 and GS-5245 significantly reduced lung titers at day 2. At day 4 post-infection, V2043, V2053, and V2067 at 27 mg/kg or 80 mg/kg significantly reduced lung titers (Fig. 4D). V2043 and V2067 both reduced lung titers to below the limit of detection in all animals, even at a V2067 dose of 8 mg/kg once-daily. EIDD-2801 also significantly reduced lung titers at 4 dpi, but obeldesivir and remdesivir did not (Fig. 4D). Vehicle-treated mice lost approximately 10% of their body weight peaking at 2 dpi (Fig. S2A and B). Compared to vehicle-treated mice, mice treated daily with V2043 80 mg/kg, V2053 27 mg/kg, and 80 mg/kg, V2067 at all doses, as well as obeldesivir 10 mg/kg or 30 mg/kg BID lost less weight at 2 dpi (Fig. S2A). Vehicle-treated mice showed mild clinical symptoms, and clinical scores did not differ (Fig. S2C). Lung histopathology was evaluated at 2 and 4 dpi using hematoxylin and eosin (H&E) staining and was not significantly different from vehicle control at 2 dpi. Mice treated with GS-5245 30 mg/kg BID and V2053 8 mg/kg had small but significant reductions in histopathology scores at 4 dpi (Fig. S3A through 3C). These studies demonstrate that oral V2043, V2053, and V2067 had robust antiviral activity in a prophylactic model of SARS-CoV-2 and were able to clear infectious SARS-CoV-2 from the lung by 4 dpi, while comparators did not.

**Fig 5:**
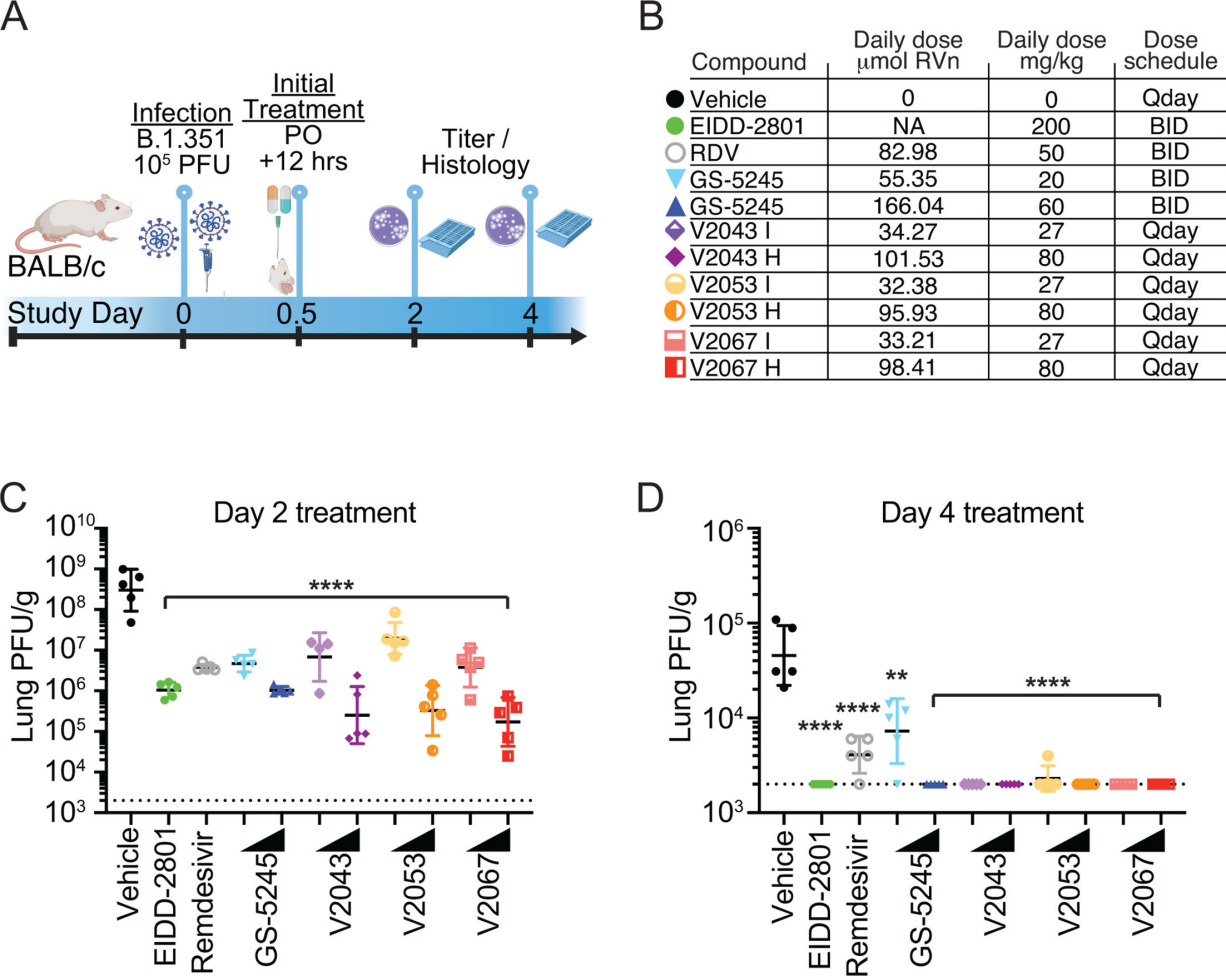
Therapeutic efficacy of oral phospholipid RVn prodrugs in a SARS-CoV-2 mouse model: (**A**) 8- to 10-week-old wild-type Balb/c mice (*n* = 5 per group/time point) were challenged with 1 × 10^5^ PFU of SARS-CoV-2 Beta variant (B.1.351). (**B**) Mice were treated with vehicle, remdesivir (GS-5734), EIDD-2801 (molnupiravir), GS-5245 (obeldesivir), or lipid RVn prodrugs V2043, V2053, or V2067 orally beginning 12 h after infection. (**C and D**) Lungs were harvested at (**C**) day 2 and (**D**) day 4 post-infection for viral titer quantification. *n* = 5 mice per group, mean ± SD is shown. Groups were compared using a mixed effect model with Geisser-Greenhouse correction with comparison of each treatment group with vehicle controls using Dunnett’s multiple comparison test; *P < 0.05, **P < 0.01, and ***P < 0.001. Dotted lines represent the PFU limit of detection.

We next evaluated phospholipid RVn prodrug efficacy in a therapeutic model of SARS-CoV-2 BALB/c infection where treatment was initiated 12 hours post-infection (hpi) (Fig. 5A). Compared to vehicle-treated mice, all drug treatments, including V2043, V2053, and V2067 at 27 mg/kg or 80 mg/kg, significantly reduced the level of infectious SARS-CoV-2 in the lung at 2 dpi (Fig. 5B and C). At 80 mg/kg once daily, V2043, V2053, and V2067 reduced lung infectious units by 3.1, 3.0, and 3.2 log_10_ at 2 dpi compared to vehicle, respectively (Fig. 5C). At 4 dpi, V2043 and V2067 at 27 mg/kg and 80 mg/kg, and V2053 at 80 mg/kg, reduced lung infectious units to below the limit of detection in all animals. EIDD-2801 100 mg/kg BID and GS-5245 30 mg/kg BID also reduced lung titers to below the limit of detection, while GS-5245 10 mg/kg BID and remdesivir 25 mg/kg BID did not. In this study, weight loss and clinical signs of disease were mild and similar in both vehicle- and compound-treated mice (Fig. 4A and B). None of the compounds significantly influenced the lung histopathology score compared to vehicle control at 2 or 4 dpi (Fig. S5A through C). Collectively, these data demonstrate that once daily oral V2043, V2053, and V2067 are potent anti-SARS-CoV-2 antivirals in both prophylaxis and treatment mouse models. Additionally, on a molar basis, V2043 and V2067 are substantially more active than GS-5245 and molnupiravir *in vivo* (Table 3).

**TABLE 3:** Molar daily dose required to reach below the limit of detection on day 4 post-infection

Compound	MW	Daily dose mg/kg	Daily dose μmoles/kg	Fold ∆ vs OBDV	Fold ∆ vs MPV
V2043	786	27	34.3	4.8×	17.7×
V2067	813	27	33.2	5.0×	18.2×
		8^*a*^	9.8	16.9×	61.9×
Obeldesivir	361	60	166	−	−
Molnupiravir	329	200	607	−	−

^*a*^ Daily dose derived from prophylaxis experiment (Fig. 4). −, not done.

## DISCUSSION

Early outpatient administration of remdesivir can significantly reduce severe COVID-19-related hospitalization and mortality. Remdesivir has remained active against all SARS-CoV-2 variants, including immune-evasive variants that are no longer susceptible to human monoclonal antibody therapies. Despite widespread clinical use, remdesivir-resistant SARS-CoV-2 variants have not emerged consistent with *in vitro* data suggesting that there is a high barrier to developing remdesivir resistance. Additionally, remdesivir is broadly active against many emerging and reemerging viruses of clinical concern. Unfortunately, the clinical utility of remdesivir outside of hospitalized patients is severely limited because it requires intravenous administration. This impedes its use early in disease, when antivirals are most effective, and restricts its use to those with access to health care facilities. An oral antiviral that can effectively deliver remdesivir triphosphate, the active metabolite, to infected tissues has the potential to be a potent broad-spectrum antiviral for the treatment of SARS-CoV-2 and many other RNA viruses.

Here, we demonstrate that two phospholipid prodrugs of remdesivir nucleoside, V2043 and V2067, are potent orally bioavailable inhibitors of SARS-CoV-2 *in vitro* and in two mouse models of infection. They are stable in plasma, active as a single oral daily dose, and 5–15× more active than obeldesivir/GS-5245 and 17–61× more active than EIDD-2801 in mouse models of SARS-CoV-2.

Collectively, the antiviral potency, PK, and efficacy data shown here demonstrate that V2043 and V2067 offer many potential benefits compared to isobutyryl ester RVn prodrugs in clinical development. Our prodrug strategy disguises RVn as partially metabolized phospholipids that are absorbed intact by the small intestine. This approach resulted in excellent oral bioavailability for V2043 and V2067 (57%–74%) in mice (Table 2). Intact phospholipid prodrugs circulate, undergo rapid cellular uptake, and enter cells before being converted to remdesivir monophosphate by a single enzymatic cleavage thereby bypassing first kinase activation (Fig. 1A). In contrast, oral isobutyryl ester prodrugs of RVn in clinical development, such as obeldesivir/GS-5245, are metabolized pre-systemically in the digestive tract (Fig. 1B). This delivers RVn to the systemic circulation where it undergoes cellular uptake and first kinase phosphorylation, both of which occur relatively slowly (Fig. 1B and C). Bypassing this initial kinase activation step contributes to the enhanced antiviral potency of V2043 and V2067 compared to RVn (Table 1). In mice, oral V2043 and V2067 administration produced sustained high-level plasma concentrations that exceeded the EC_90_ of SARS-CoV-2 for more than 24 h. The daily systemic exposure of V2043 and V2067 after a once daily oral 60 mg/kg dose in BALB/c mice was 199 and 154 µM*hour, respectively (Table 2). In comparison, a recent study showed that the daily systemic exposure of obeldesivir/GS-5245 after twice daily oral 30 mg/kg doses in BALB/c mice was 108 µM*hour (10). Thus, oral V2043 and V2067 appear to produce higher daily systemic exposures than obeldesivir/GS-5245 even when administered once daily and at lower molar doses, 73 and 76 µmol/kg/day compared to 166 µmol/kg/day. Consistent with the excellent *in vitro* potency and promising PK of V2043 and V2067, both treatments effectively reduced SARS-CoV-2 titers in both prophylaxis and therapeutic mouse models of SARS-CoV-2 infection (Fig. 4 and 5). Importantly, at least 5-fold or 17-fold lower molar doses of V2043 and V2067 were needed to reduce infectious virus titers below the limit of detection at day 4 post-infection, compared to GS-5245 or molnupiravir, respectively, suggesting that V2043 and V2067 are more potent antivirals *in vivo* (Table 3). Due to its structural complexity, RVn is challenging and expensive to manufacture (11). Developing potent prodrugs of RVn, such as V2043 and V2067, could reduce costs by significantly reducing the required dose needed for treatment.

While V2053 showed excellent *in vitro* activity, its less favorable pharmacokinetic profile likely contributed to its reduced *in vivo* efficacy compared to V2043 and V2067. Understanding the relationships of R_1_ and R_2_ groups on the pharmacokinetics of phospholipid prodrugs independently of their effects on cell entry or conversion to the active metabolite will likely lead to further improvements.

In addition to anti-coronavirus activity, we have previously shown that V2043 and V2067 have excellent *in vitro* antiviral activity against many clinically important RNA viruses, including respiratory syncytial virus, emerging henipaviruses, and flaviviruses (8, 12). Evaluation of the *in vivo* efficacy of V2043 and V2067 against additional RNA viruses is needed to understand their potential as oral broad-spectrum RNA virus inhibitors.

In conclusion, V2043 and 2067 are potent orally bioavailable phospholipid prodrugs of RVn that may have clinical utility in the treatment of SARS-CoV-2 and other RNA viruses of clinical importance. These data support the continued development of phospholipid RVn prodrugs and their evaluation for safety and dose finding in humans.

## MATERIALS AND METHODS

### Compounds

V2043 was synthesized at Nanosyn (Santa Clara, CA, 99.3% HPLC purity). V2053 and V2067 were synthesized at J-Star Research (South Plainfield, NJ, 98.8% and 98.7% HPLC purity, respectively). Obeldesivir (GS-5245) was synthesized at University of California, San Diego, as described in Mackman, Richard L., et al. (11). The structure was confirmed using ^1^H NMR and mass spectroscopy, and the EC_50_ (1.196 ± 0.084—Table 1) of the compound was similar to published data when using a similar model of SARS-CoV-2 infection in A549-hACE2-hTMPRSS2 (11). Remdesivir and EIDD-2801 (molnupiravir) were purchased from MedChem Express (Monmouth Junction, NJ).

### Viruses and cells

To prepare the virus for animal studies, cells and viruses were handled and processed as published previously. Vero-E6 cells (ATCC# CRL 1586) were cultured in DMEM (Quality Biological), with 10% (vol/vol) FBS (Gibco), 1% (vol/vol) penicillin/streptomycin (Gemini Bio-Products), and 1% (vol/vol) L-glutamine (Gibco). Cells were grown at 37°C with 5% CO_2_. Virus stocks were amplified using the ARTIC primer set and sequenced using the MinION system (Oxford Nanopore Technologies) by the J. Craig Venter Institute (MD, USA) to more than 4,000× genome coverage. The stock sequence was verified by aligning reads to the reference genome provided by the BEI (Beta variant GISAID accession: EPI_ISL_890360). Stocks had a less than 1% variation. Media were collected and clarified by centrifugation before being aliquoted for storage at −80°C. Titer of stock was determined by plaque assay using Vero-E6 cells expressing TMPRSS2. All work with infectious viruses was performed in a biosafety level 3 laboratory and approved by the University of Maryland School of Medicine Institutional Biosafety Committee.

For *in vitro* antiviral assays (Table 1), cell lines Vero-E6-TMPRSS2 (Sekisui XenoTech), Calu-3 (ATCC #HTB-55), Huh7.5 (Apath LLC), and A549-ACE2/TMPRSS2 (A2T2-A549) (ATCC #CRL-3560) were cultured at 37°C and 5% CO_2_. Vero-E6-TMPRSS2 were grown in DMEM (Corning, Cat. #10-013-CV) plus 10% FBS (Biowest, Cat. #S1620), 1% (vol/vol) penicillin/streptomycin (Gibco, Cat. #15140-122), and 1 mg/mL geneticin (Gibco, Cat. #10131027). Calu-3 cells were grown in MEM (Gibco, Cat. #11090099) plus 10% FBS, 1% (vol/vol) penicillin/streptomycin, 1 mM sodium pyruvate (Gibco, Cat. #11360070), and 1% (vol/vol) GlutaMAX (Gibco, Cat. #35050061). Huh7.5 and A549-A2T2 cells were grown in DMEM plus 10% FBS and 1% (vol/vol) penicillin/streptomycin. SARS-CoV-2 WA1 was acquired from BEI (NR-52281), passaged once through Caco2 cells (ATCC), expanded on Vero-E6-TMPRSS2 cells, and verified by whole-genome sequencing. Virus stocks were titered by fluorescent focus assay on Vero-E6-TMPRSS2 cells. Experiments with infectious SARS-CoV-2 isolate WA1 were conducted at University of California San Diego under BSL3 conditions as approved by the Institutional Biosafety Committee.

### *In vitro* antiviral assays

Cells were pretreated 30–60 min with threefold dilutions of compounds in a complete growth medium and infected at MOIs of 0.013 for Vero-TMPRSS2, 0.02 for Calu-3, and 0.1 for Huh7.5. Cells were incubated in the presence of virus and compounds for 32 h for Vero-TMPRSS2, 44–48h for Calu-3, and 48 h for Huh7.5 and A2T2-A549 cells before fixation with 4% formaldehyde for 30 min at RT. Infected cells were stained with anti-nucleocapsid primary antibody (GeneTex, Cat. #gtx135357), anti-rabbit AlexaFluor 594 secondary antibody (ThermoFisher Scientific, Cat. # A11037), and Sytox Green nuclear counterstain (ThermoFisher Scientific, Cat. #S7020). Five images per well were acquired at 10× magnification on an Incucyte SX5, and percent infection was calculated by automatically detecting total nuclei and nucleocapsid-positive cells using the Incucyte image analysis software. Relative infection was determined by normalizing to DMSO control wells on each plate. Best fit curves, EC_50_, and EC_90_ were calculated in Prism 10.

### Cell viability assays

Cell viability assays (Table 1) were performed as previously described (7). Cells were cultured and seeded as per *in vitro* antiviral studies in opaque walled 96-well cell culture plates and incubated overnight for Huh7.5, A2T2-A549, and Vero-E6-TMPRSS2 cells and for 2 days for Calu-3 cells. Compounds or controls were added at the indicated concentrations in a total volume of 100 µL, and cells were incubated for 48 h. An equal volume of CellTiter-Glo reagent (Promega, Cat. #G7570) or CellTiter-Glo 2.0 reagent (Promega, Cat. #G9241) was added and mixed, and luminescence was recorded on a Veritas Microplate Luminometer (Turner BioSystems) or a Spark Multimode plate reader (Tecan) according to manufacturer instructions. Percent viability was normalized to DMSO controls, and CC_50_ values were derived from best fit curves calculated in Prism 10.

### Analysis of prodrugs in plasma

Phospholipid RVn prodrugs V2043, V2053, and V2067 were analyzed in plasma samples by a methanol protein crash and quantitated against a freshly prepared calibration curve using a bestfit regression from 1.0 to 1,000 ng/mL for the prodrugs. After fortification, 50 µL of plasma was crashed with 400 µL of methanol containing 10 ng/mL of the internal standard (V2066). One hundred fifty microliters of the supernatant was removed and diluted with 150 µL of water in a clean 96 well injection plate.

Chromatography for the prodrugs was performed on a Raptor Biphenyl (2.1 × 50) column at a flow rate of 0.5 mL/min. A gradient method starting at 60% B (0.1% formic acid in 90:10 methanol:acetonitrile) for 30 s and then ramping to 95% B over 2.5 min. This composition was held for 1.0 min before being returned to starting conditions and re-equilibration to 40% A (10 mM ammonium formate with 0.2% formic acid in water). The peak area ratio (analyte/internal standard) for V2043 (786.4 → 513.4), V2053 (832.4 → 559.4), V2067 (811.4 → 538.4), and the internal standard (584.4 → 581.4) were plotted against the standard concentrations (*X* axis) and a best fit regression (linear or quadratic) was used to quantitate the amount of prodrug in the samples.

### Animal studies

The University of Maryland School of Medicine is accredited by the Association for Assessment and Accreditation of Laboratory Animal Care (AAALAC International). All animal procedures were done in accordance with the NRC (National Research Council) Guide for the Care and Use of Laboratory Animals, the NIH (National Institutes of Health)/CDC (Centers for Disease Control and Prevention) Biosafety Guidelines in Microbiological and Biomedical Laboratories, and the Animal Welfare Act. All mouse studies were approved by the University of Maryland School of Medicine Institute for Animal Care and Use Committee. Studies were done in accordance with the NIH Health Guide for Care and Use of Laboratory Animals (NIH publication 8023, revised 1978). All mouse infections were carried out in an animal biosafety level 3 facility in accordance with pre-approved practices.

Oral pharmacokinetic studies were carried out at *In Vivo* Technologies (Moffatt Field, CA) in male BALB/c mice, 22–23 g under IACUC protocol number IVT-11–010Y12 (LifeSource Animal Care and Use Committee). V2043, V2053, and V2067 were administered by gavage in 0.1 M sodium carbonate/bicarbonate, pH 9, at the indicated doses. Blood was obtained by facial vein or terminal cardiac puncture at the indicated times and plasma was frozen for analysis. Similar procedures were followed for 5-day tolerability and terminal dose pharmacokinetic studies in mice at oral daily doses of 80 or 100 mg/kg. In the 80 and 100 mg/kg tolerability study, no adverse clinical effects were noted and animal weights on day 5 in the treated animals were similar to those in a vehicle control.

A 7-day oral tolerability and terminal pharmacokinetic study in 100–125 g male Syrian hamsters were done at Attentive Sciences (Stillwell, KS), under IACUC approval number 1123–6788. V2043 in 0.1 M sodium carbonate/bicarbonate, pH 9, was administered by oral gavage in a daily dose of 100 mg/kg. Daily clinical observation and weights were obtained. After the day 7 oral dose blood was obtained at the indicated times and plasma was frozen for analysis. No ill effects were reported, and day 7 weights were similar to an untreated control group.

### Mouse infections

Eight- to 10-week-old female BALB/ were purchased from Jackson Laboratories. On day 0, mice were given an intraperitoneal injection of 50 µL mix of ketamine (1.3 mg/mouse) and xylazine (0.38 mg/mouse) diluted together in phosphate-buffered saline (PBS). While anesthetized, mice were intranasally inoculated with either 50 µL of sterile PBS or virus. The virus used was the B.1.351 Beta variant of SARS-CoV-2.

### Drug administration

Mice were given an oral gavage volume of 160 µL per drug administration. For EIDD-2801, the drug was prepared in 10% DMSO (dimethyl sulfoxide) (2438, Sigma) and 90% corn oil (8627, Sigma) and administered twice daily. For Remdesivir, the drug was prepared in 10% DMSO and 90% corn oil and administered twice daily. GS-5245 was prepared in water and administered twice daily. The three lipid remdesivir nucleoside prodrugs, V2043, V2053, and V2067, were prepared in 0.1 M sodium carbonate/bicarbonate buffer pH 9 and administered once daily. The carrier-only group received 0.1 M sodium carbonate/bicarbonate buffer pH 9 alone twice daily. For prophylactic dosing, drugs were administered beginning 12 h before infection. For therapeutic dosing, drugs were administered 12 h after infection.

### Histology and immunohistochemistry

Mouse lungs were fixed in 4% paraformaldehyde (PFA) in PBS for at least 48 h. Fixed lungs were sent to the University of Maryland Baltimore Histology core facility for paraffin embedding, sectioning into 5 µm sections, and staining with hematoxylin and eosin staining. Lungs were scored in a blinded fashion with a 0 to 5 score given, 0 being no inflammation and 5 being the highest degree of inflammation. Interstitial inflammation and peribronchiolar inflammation were scored separately. Scores were then averaged for the overall inflammation score.

### Virus titers/plaque assay

Vero-E6/TMPRSS2 cells were cultured in DMEM (Dulbecco’s modified Eagle medium) (Quality Biological), supplemented with 10% FBS (Sigma), 1% (vol/vol) penicillin/streptomycin (Gemini Bio-products), and 1% (vol/vol) L-glutamine (Gibco). Cells were grown and maintained in an incubator at 37°C and 5% CO2. Viral lung titers were quantified by homogenizing lung tissues in PBS (Quality Biological) with 1.0 mm glass beads (Sigma) in a Beadraptor (Omni International). Vero-E6 cells were plated in 12-well plates with 2.0 × 10^5^ cells per well. Plaque assay was performed by adding 25 µL lung homogenate after centrifugation to 225 µL DMEM with 10-fold dilutions across a six-point dilution curve with 200 µL DMEM diluent added to each of the wells. After a 1 h dilution with plate rocking every 15 min, a 2 mL agar overlay containing DMEM is added to each of the wells. Plates are incubated for 2 days at 37°C and 5% CO2. Then, plaques are fixed with 10% formalin, stained with crystal violet, washed with tap water, and counted.

### Clinical scoring

Clinical signs of disease were assessed daily in mice. Clinical scores were determined on the following scale: 0 = healthy; 1 = slight ruffling of the fur, altered hind limb posture; 2 = mildly labored breathing, no lethargy, 3 = moderately labored breathing, lethargy; 4 = severely labored breathing, severe lethargy; and 5 = dead.

### Statistical analyses

Statistics were performed with GraphPad Prism 10.2.1 software (GraphPad Software, San Diego, CA). Data were analyzed using unpaired *t*-test, one- or two-way analysis of variance (ANOVA) followed by Tukey, Dunnett, or Sidak *post hoc* comparison test as indicated. All statistical analyses were two-sided and a *P* < 0.05 was considered statistically significant.
